# Predicted Functional Potentials of Bacterial Communities in Fermented Maize Products From Ghana, Nigeria, and Benin via 16S rRNA Amplicon Sequencing and PICRUSt2

**DOI:** 10.1002/mbo3.70272

**Published:** 2026-06-26

**Authors:** Humphrey P. K. Addy, David Amedorme, Priscilla Osei‐Poku, Alexander Kwarteng

**Affiliations:** ^1^ Department of Biochemistry and Biotechnology, College of Science Kwame Nkrumah University of Science and Technology Kumasi Ghana; ^2^ Department of Biomedical Sciences, School of Allied Health Sciences University of Cape Coast Cape Coast Ghana; ^3^ Council for Scientific and Industrial Research Animal Research Institute Accra Ghana; ^4^ Kumasi Centre for Collaborative Research in Tropical Medicine Kwame Nkrumah University of Science and Technology Kumasi Ghana

**Keywords:** acetobacter, amplicon sequencing, DNA‐Hhelicase, fermented foods, firmicutes, lactobacillus, predictive metagenomics

## Abstract

Fermented maize products are integral to the diets of many African communities. Despite their cultural significance and health benefits, little is known about the metabolic potential of their microbial populations. This study utilized 16S rRNA amplicon sequencing data from the NCBI to characterize the functional capabilities of microbiomes in six maize‐based fermented foods. Quality assessment and taxonomic classification were performed using QIIME2 with the SILVA 138 database, while functional predictions were generated with PICRUSt2 and analyzed in R. Taxonomic profiling revealed that *Firmicutes* dominated all samples, reaching peak abundance in Mawe (94.9%) and S37_Fermented_Maize (91.4%). *Proteobacteria* were elevated in S19_Fermented_maize (up to 36.5%) and S38_Dehulled_Maize (16.0%). At the genus level, *Lactobacillus* was most abundant in S5_Mawe (82.2%) and S6_Mawe (79.6%), while *Acetobacter* peaked in S19_Fermented_maize (32.7%). Regarding functional predictions, *Lactobacillus* appeared to drive key KEGG Orthologs and pathways, specifically ABC transporters, transcriptional regulation, and DNA replication mechanisms. In contrast, *Weissella* and *Streptococcus* contributed notably to peptide/nickel transport, L‐lactate dehydrogenase (EC 1.1.1.27), and nucleotide biosynthesis. *Acetobacter* was prominent in Ogi, showing a connection with site‐specific methylation (EC 2.1.1.72) and phospholipid synthesis (PHOSLIPSYN‐PWY). Notably, commercial Mawe samples exhibited higher predicted activities related to transposase activity (K07496), energy metabolism, and peptidoglycan maturation (PWY0‐1586). These findings demonstrate that while traditional fermentation processes maintain a consistent set of metabolic functions predominantly driven by *Lactobacillus*, distinct variations exist depending on product type and production approach. These predicted functions provide a baseline for further experimental validation of the metabolic contributions of microbial communities in fermented maize products.

## Graphical abstract

Publicly available 16S rRNA data from West African fermented maize products were analyzed in QIIME2, profiled with SILVA 138, and functionally predicted using PICRUSt2, revealing *Lactobacillus*‐dominated microbiomes driving key metabolic pathways in carbohydrate utilization, amino acid metabolism, DNA replication, and cell wall biosynthesis.

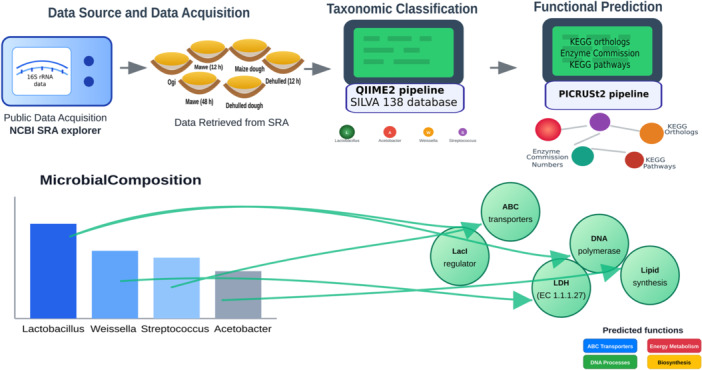

## 1 Introduction

Fermented maize products are a fundamental part of diets in regions where maize serves as a staple food, offering both cultural significance and important nutritional benefits. These foods, produced through lactic acid fermentation, undergo complex biochemical transformations driven by diverse microbial communities (Atter et al. 2024). These microbial consortia, which are commonly dominated by lactic acid bacteria (LAB) (Tamang et al. 2020), alter the physicochemical properties of maize substrates, enhancing food safety by inhibiting spoilage organisms, improving palatability, and increasing the bioavailability of nutrients such as vitamins, amino acids, and minerals (López‐Sánchez et al. 2023; Madilo et al. 2024; Obafemi et al. 2022). Additionally, this microbial activity not only helps reduce anti‐nutritional factors like phytic acid and polyphenols, which typically limit nutrient absorption, but also promotes the synthesis and release of bioactive compounds, including vitamins (notably B‐complex vitamins like thiamine, riboflavin, and niacin) and potentially beneficial peptides and enzymes. These changes lead to improved digestibility and nutritional quality of maize‐based fermented products such as Ogi, kenkey, and mahewu, which are consumed widely across Africa (Atter et al. 2021; Shingling and Tamang 2021).

However, a research gap exists in our understanding of these systems. While the taxonomic composition of African fermented foods has been documented, the majority of existing studies rely on culture‐dependent methods or basic amplicon sequencing that describes who is there, without adequately exploring what they are doing (De Filippis et al. 2018; Madilo et al. 2024). There is a scarcity of data linking specific taxonomic clusters to the metabolic potential that drives the unique organoleptic and nutritional profiles of West African maize products. Understanding the functional capacity of these microbiomes is essential for standardizing production and maximizing health benefits.

To address this, we employed 16S rRNA amplicon sequencing combined with predictive metagenomics (PICRUSt2) to analyze microbial dynamics in traditional maize‐based fermented products from Ghana, Benin, and Nigeria. Beyond traditional descriptions of diversity, this approach allows for the *in silico* reconstruction of metabolic pathways, offering insights into functional roles related to fermentation dynamics, nutritional enhancement, and food safety, that are difficult to study through cultivation alone (Douglas et al. 2019; Umokaso et al. 2022; Mukherjee et al. 2022).

We hypothesized that distinct traditional processing methods, such as cooking and raw dough processing, exert selective pressure that drives the convergence of a core functional microbiome characterized by specific metabolic pathways for carbohydrate metabolism and stress adaptation, regardless of geographic origin.

By characterizing these functional signatures, this study aims to provide a predictive baseline for the metabolic capabilities of fermentation microbiota. Here, we show patterns of traditional production or processing methods and how they exert selective pressure that drives the convergence of a core functional microbiome, characterized by specific metabolic pathways for carbohydrate metabolism and stress adaptation, regardless of geographic origin. Integrating these ecological and functional insights supports efforts toward quality control, nutritional valorization, and the development of targeted microbial consortia, which are essential steps for improving the consistency and value of these culturally important foods.

## 2 Methods

### 2.1 Data Acquisition and Data Source

This study leveraged publicly accessible 16S rRNA amplicon sequencing datasets derived from traditional maize‐based fermented foods. These datasets were obtained from the National Center for Biotechnology Information (NCBI) database. A systematic search was conducted on July 1, 2025 using targeted keywords including “fermented foods and 16S and cereals” (https://www.ncbi.nlm.nih.gov/search/all/?term=fermented%20foods%20and%2016S%20and%20cereals), “african fermented foods and 16S and cereals” (https://www.ncbi.nlm.nih.gov/search/all/?term=african%20fermented%20foods%20and%2016S%20and%20cereals), and “african fermented foods and amplicon sequencing and cereals”(https://www.ncbi.nlm.nih.gov/search/all/?term=african%20fermented%20foods%20and%20amplicon%20sequencing%20and%20cereals). These queries identified a single relevant BioProject (accession number PRJNA532858), corresponding to the cross‐sectional study conducted by Diaz et al. (2019).

**Figure 1:**
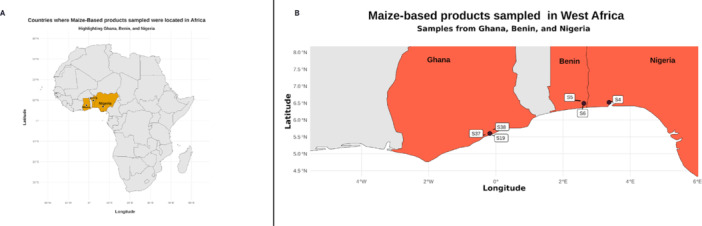
The geographic origin of the maize products selected for this study. Panel (a) displays a map of Africa highlighting (in golden yellow), the three countries from which the maize samples were sourced in the original study. Panel (b) provides a zoomed‐in view of specific locations within these countries (in coral).

This study focused on maize‐derived samples sourced from West African food systems to ensure relevance to traditional fermentation practices in this region. Samples were selected from the dataset reported by Diaz et al. (2019), which originally included maize product samples collected from three West African countries: Ghana, Benin, and Nigeria (Figure 1). The original dataset was generated using a cross‐sectional sampling design within a defined timeframe, thereby minimizing temporal biases such as seasonal variation. Metadata for each sample were curated from the Sequence Read Archive (SRA) Explorer, SRA Run Selector, and the original publication, providing comprehensive contextual information for downstream analyses.

### 2.2 Sample Selection

Six maize‐based samples representing diverse fermentation statuses, processing methods, and production conditions were selected to capture the functional variability characteristic of traditional fermented maize products in West Africa (Table 1). The samples included one artisanal cooked fermented product, Ogi (S4) from Nigeria; two commercially produced Mawe samples (S5 and S6) from Benin subjected to 12‐h and 48‐h fermentation, respectively; and three maize dough variants from Ghana (S19, S37, and S38) differing in fermentation, dehulling, and cooking status.

**Table 1:** Summary of the background data of selected 16S raw reads for corresponding maize products.

ID	Run	Source	Cooked/processed	Production conditions	Country
S4	SRR8901481	Ogi from maize	Yes	Artisanal	Nigeria
S5	SRR8901482	Mawe (12 h fermented)	Yes	Commercial	Benin
S6	SRR8901483	Mawe (48 h fermented)	Yes	Commercial	Benin
S19	SRR8901464	Maize dough	No	Artisanal	Ghana
S37	SRR8901463	Dehulled maize dough	No	Commercial	Ghana
S38	SRR8901489	Dehulled maize dough (12 h fermented)	No	Commercial	Ghana

Samples were obtained from varied geographical locations within West Africa. The selection criteria aimed to represent key variables influencing microbial and enzymatic functions, including fermentation duration (unfermented, 12 h, 48 h), processing (cooked vs. raw), and production mode (artisanal vs. commercial). This strategic selection enables comprehensive functional profiling across fermentation states, processing methods, and production practices, providing insights into the microbial and enzymatic landscape shaped by traditional maize fermentation.

### 2.3 Data Retrieval

The raw sequencing data utilized in this analysis was retrieved from the NCBI Sequence Read Archive (SRA) under BioProject accession PRJNA532858. Originally generated by Diaz et al. (2019) as part of the “Analysis of the Microbiomes of Naturally Fermented Foods” training workshop, the dataset comprises 16S rRNA amplicon sequences from 40 African fermented food samples, including those based on maize, which are central to the present study, as shown in Table 1.

This original study received funding from the UK Biotechnology and Biological Sciences Research Council (BBSRC) through the Global Challenge Research Fund Data and Resources award and Institute Strategic Programmes for Food Innovation and Health (BB/R012512/1) and other projects BBS/E/F/000PR10343 and Gut Microbes and Health (BB/R012490/1).

### 2.4 Bioinformatics Analysis

#### 2.4.1 Data Quality Control and Processing

The paired‐end raw reads were screened for quality control metrics, while confirming that all sequencing adapters were removed, and quality parameters were good for downstream analysis using fastQC and multiQC. The paired‐end reads were imported into QIIME2 amplicon sequencing analysis tool (v2024.2) for denoising using the DADA2 plugin for the purposes of low‐quality reads filtering, error‐correction, chimera removal, and to generate representative sequences for taxonomy classification based on amplicon sequence variants (ASVs) (Callahan et al. 2019; Srivastava et al. 2024).

#### 2.4.2 Taxonomy Classification and Functional Predictions

Taxonomic classification of the ASVs was computed using the Naïve Bayes classifier plugin in QIIME2 (v2024.2) pipeline (Estaki et al. 2020; Hall and Beiko 2018), which was trained on the SILVA 138 database at a similarity threshold of 99% on the V4 hypervariable region of the 16S rRNA. Additionally, phylogenetic analysis was executed through the MAFFT‐FastTree plugin (Estaki et al. 2020). The generated taxonomic tables and phylogenetic trees were exported for subsequent analyses in the R software (v4.4.2), using the Phyloseq (v1.48.0) and qiime2R (v0.99.6) packages. We predicted the gene content of all ASVs by customizing the default settings with an option *picrust2_pipeline. py ‐‐stratified* to account for specific ASV contributions to predicted functions. The output datasets for predicted enzymes based on enzyme commission numbers, predicted KEGG orthologs, and predicted KEGG pathways were adopted for downstream analysis and visualization in RStudio running R v4.3.2.

#### 2.4.3 Functional Data Analysis and Visualization in R

The R software was used to explore predicted microbial functions from the PICRUSt2 outputs. Using readr (v1.4.3), we imported three datasets containing the relative abundance values for all samples for downstream analysis. These datasets which are available in the supporting materials (S1‐S3), were reshaped into long format using dplyr (v1.1.4) and tidyr (v1.3.1) to support comparative visualization. Features were ranked by their combined relative abundance across both samples, and the top 10 in terms of relative abundance in each category of predicted EC numbers, KO gene families, and KEGG pathways were selected for detailed graphical representations. Bar plots were generated using ggplot2 (v3.5.1) to visually distinguish between sample types and enhance consistency and readability. To enable comparative pattern recognition, we constructed clustered heatmaps using the pheatmap (v1.0.12) package. Prior to heatmap construction, the relative abundance following specific taxa contributions to each of the functions in the PICRUSt datasets was maintained to preserve analytical coherence. These visualizations provide an overview of shared and unique metabolic traits associated with the bacterial communities in all samples, facilitating a descriptive interpretation of their predicted functional repertoires.

## 3 Results

The study analyzed six maize‐derived samples representing various fermentation statuses, processing methods, and production conditions across West Africa.

### 3.1 Taxonomic Composition of Microbial Communities

#### 3.1.1 Taxonomic Composition at the Phyla Level

**Figure 2:**
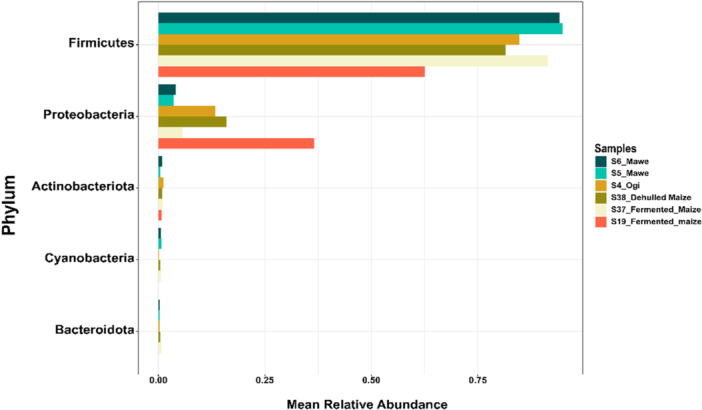
Dominant taxa and relative abundance of represented Phyla.

**Figure 3:**
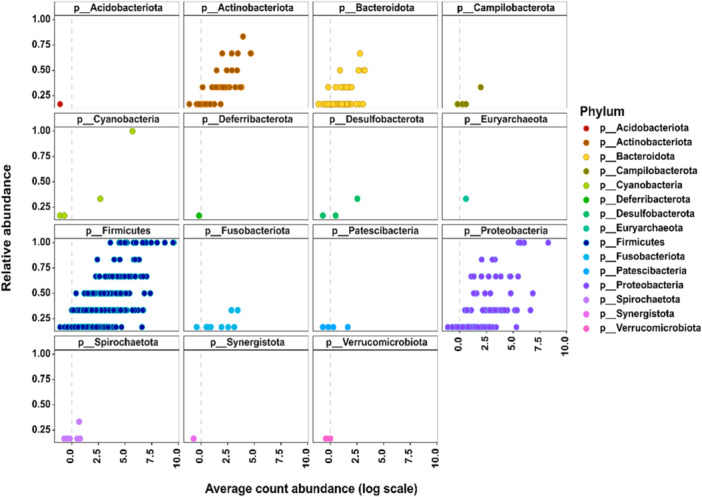
Taxonomic composition and relative abundance of represented Phyla.

From the taxonomic classification leveraging the SILVA 138 database, it showed a diverse array of prokaryotic communities in the six fermented maize‐based food samples at the phylum, down to the genus and species levels. At the phylum level, *Firmicutes* dominated all samples, with the highest proportions in S5_Mawe (*n* = 72,640; 94.9%) and S37_Fermented_Maiz (*n* = 72,428; 91.4%) (Figure 2). *Proteobacteria* were prevalent in S19_Fermented_maize (*n* = 27,175; 36.5%) and S38_Dehulled_Maize (*n* = 11,295; 16.0%). *Actinobacteriota* exhibited a stable low‐level abundance across all samples, peaking in S4_Ogi (*n* = 839; 1.2%), while *Cyanobacteria* and *Fusobacteriota* appeared sporadically, as shown in Figure 3.

#### 3.1.2 Taxonomic Composition at the Family Level

**Figure 4:**
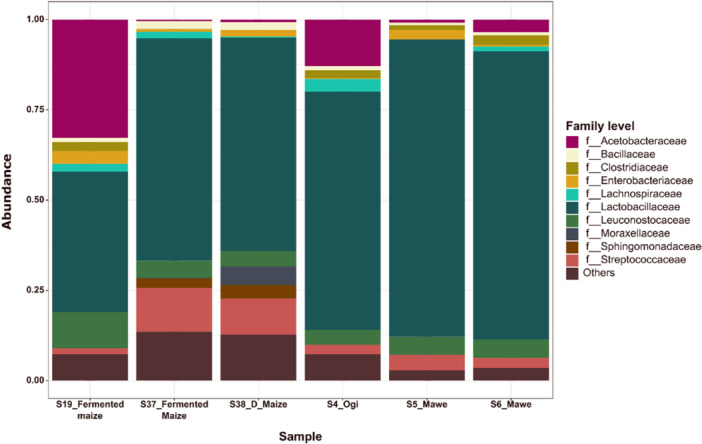
Taxonomic composition and relative abundance at the Family level.

*Lactobacillaceae* predominated in all products at the family level, especially in S5_Mawe (*n* = 63,051; 82.4%) and S6_Mawe (n = 57,861; 79.8%). *Acetobacteraceae* was relatively abundant in S19_Fermented_maize (*n* = 24,338; 32.7%) showed a lower abundance in S37_Fermented_Maiz (*n* = 372; 0.5%). Other families included *Streptococcaceae* in S37_Fermented_Maiz (*n* = 9716; 12.3%) and S5_Mawe (*n* = 3285; 4.3%), as well as *Moraxellaceae* and *Erysipelotrichaceae* in S38_Dehulled_Maize and S37_Fermented_Maiz, respectively, as shown in Figure 4.

#### 3.1.3 Taxonomic Composition at the Genus Level

**Figure 5:**
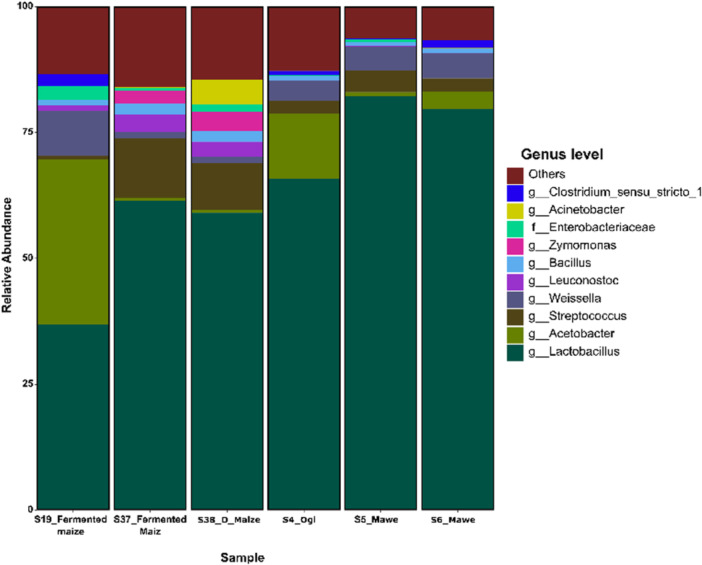
Taxonomic composition and relative abundance of predominant Genera.

The genus level patterns showed the dominance of *Lactobacillus* in S5_Mawe (*n* = 62,922; 82.2%) and S6_Mawe (*n* = 57,730; 79.6%). *Acetobacter* was enriched in S19_Fermented_maize (*n* = 24,305; 32.7%), while *Weissella* was present in both S19_Fermented_maize and S5_Mawe. As shown in Figure 5, niche specialists, *Zymomonas* and *Acinetobacter* were observed in the dehulled maize and the S37 maize dough samples.

#### 3.1.4 Taxonomic Composition at the Species Level

At the species level, *Lactobacillus fermentum* was the most abundant taxon in S37_Fermented_Maiz (*n* = 10,585; 42.0%) and S38_Dehulled_Maize (*n* = 10,916; 42.3%). Other species observed include *Zymomonas mobilis* and *Lactobacillus sanfranciscensis*. Diversity in S19_Fermented_maize included *Lactobacillus amylovorus*, *L. delbrueckii*, *L. Helveticus*, among others. S4_Ogi was characterized by the dominance of *L. delbrueckii* (40.3%) and proportions of *Acetobacter lovaniensis* and *Clostridium perfringens*. The Mawe samples showed the highest diversity, including *L. Pontis*, *L. Helveticus*, *L. brevis*, *L. Reuteri* and *L. Mesenteroides*. The data supporting these observations are provided in the Supplementary Material (Supplementary File S4).

### 3.2 Microbial Community Diversity

**Figure 6:**
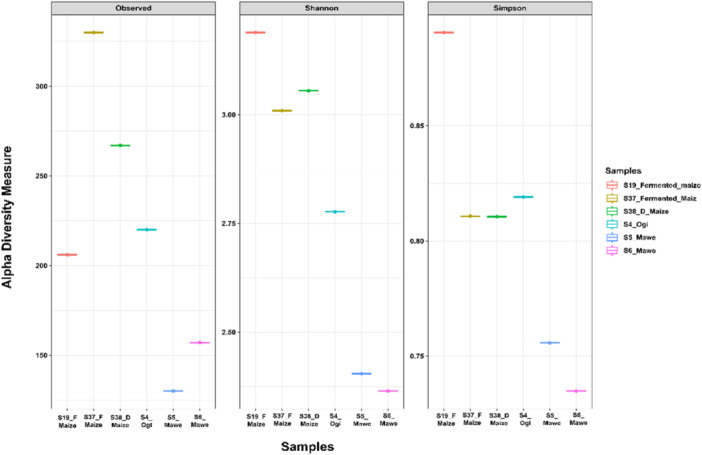
Alpha Diversity metrics for all samples.

**Figure 7:**
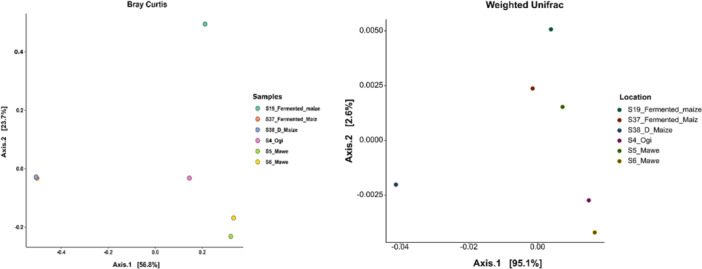
Beta Diversity metrics (Bray–Curtis and Weighted UniFrac) for all samples.

Alpha diversity metrics, as illustrated in Figure 6, showed that S37_Fermented_Maiz had the highest richness (330 ASVs), while S5_Mawe had the lowest (130 ASVs). S19_Fermented_maize had the highest Shannon and Simpson indices (3.19 and 0.89), indicating high diversity and evenness. The beta diversity analysis using Bray‐Curtis and Weighted UniFrac metrics, as shown in Figure 7, revealed distinctions in microbial composition, with Principal Coordinates Analysis (PCoA) plots separating S37_Fermented_Maize and S38_Dehulled_Maize from S5_Mawe and S6_Mawe.

### 3.3 Functional Prediction of Microbial Communities: KEGG Genes, Enzyme Commissions, and KEGG Pathways

#### 3.3.1 Functional Predictions of KEGG Orthologs

**Figure 8:**
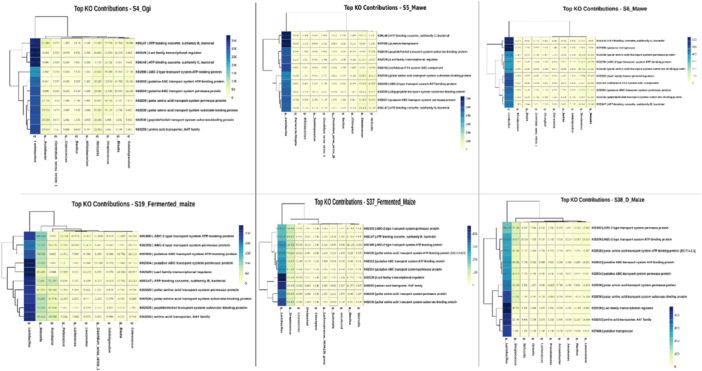
Combined heatmaps showing contributions of the top 10 bacterial genera to KEGG Orthologs across six maize samples.

Our PICRUSt2 analysis yielded multiple KEGG Orthologs (KOs) in six (6) fermented maize‐based food samples, revealing functions with high abundance and consistent presence across the samples. From the composite heatmaps as shown in Figure 8, *Lactobacillus* was the predominant contributor to the functional roles of most KOs across all six samples, whereas the contributions from other genera exhibited variability depending on the sample type. In S19 (fermented maize dough), *Lactobacillus* demonstrated the highest relative abundance among all major KOs, including K02029 (polar amino acid transport permease; 251.21) and K02529 (LacI transcriptional regulator; 289.08). In contrast, *Weissella* was associated with K01990 (ABC‐2 ATP‐binding protein; 106.36) and K02035 (peptide/nickel transport system ATP‐binding protein; 79.57). *Lactobacillus* was predominant in S37 (dehulled maize dough), particularly in K02529 (461.48) and K02029 (379.26). *Streptococcus* demonstrated notable levels in K01990 (115.36) and K06147 (ABC transporter, subfamily B; 99.04). S38 (12h‐fermented dehulled dough) demonstrated significant KO contributions from *Lactobacillus*, notably for K02529 (464.89) and K02029 (387.14). *Streptococcus* represented 93.68% of K01990, whereas *Weissella* contributed 6.45% to K03293 (amino acid transporter). S4 (Ogi) demonstrated notable contributions from *Lactobacillus* for K06148 (ABC transporter, subfamily C; 368.75) and K03293 (amino acid transporter; 309.94), while *Acetobacter* contributed to K03293 (26.65) and K02029 (23.31). S5 (12h‐fermented Mawe) showed a predominance of *Lactobacillus*, with K07496 (transposase; 627.21), K02529 (582.75), and K06148 (548.29) identified as the most prevalent sequences. *Weissella* contributed 42.83 to K01990 and 37.62 to K02035. *Lactobacillus* was the predominant genus in K07496 (634.05), K06148 (602.28), and K02529 (587.42) for S6 (48‐h fermented mawe, commercial), while *Weissella* was present in K01990 (42.16) and K02035 (38.57).

**Figure 9:**
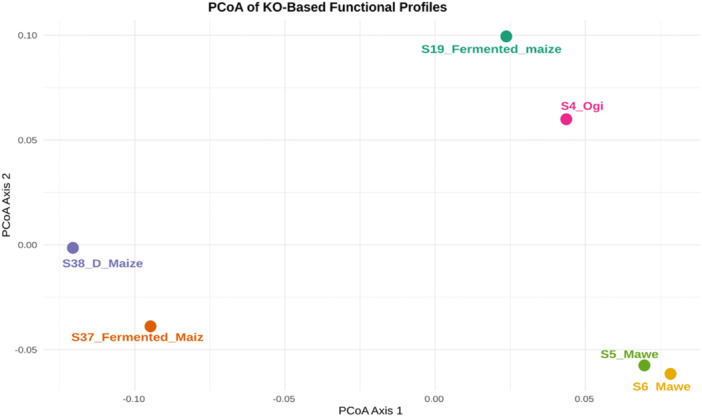
PCoA of KEGG Ortholog (KO) contributions across samples.

From the PCoA plot based on the predicted KEGG Orthologs, the analysis revealed distinct clustering patterns corresponding to each sample type, highlighting significant functional differentiation (Figure 9). Notably, S5_Mawe and S6_Mawe samples clustered closely. In contrast, S19_Fermented_maize and S37_Fermented_Maize, despite sharing the product category, exhibited notable separation, whilst S38_D_Maize appeared distinctly separated from all fermented counterparts, emphasizing the profound functional shifts induced by fermentation. S4_Ogi presented a unique profile, distinct from both fermented maize and Mawe, emphasizing product‐specific microbial functionalities.

#### 3.3.2 Functional Predictions of KEGG Enzyme Commission

**Figure 10:**
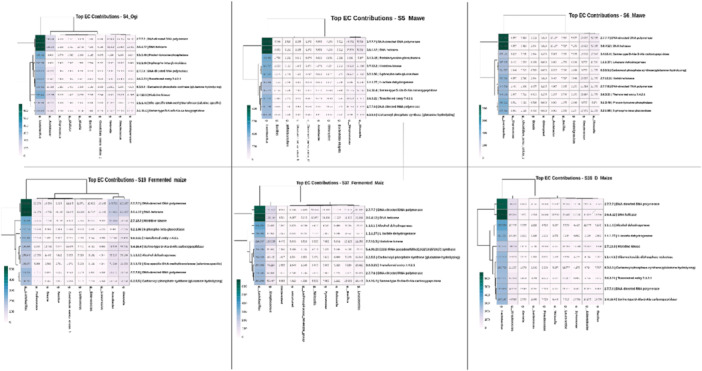
Combined heatmaps showing contributions of the top 10 bacterial genera to Enzyme Commission (EC) functions across six maize samples.

The functional predictions at the EC level indicated significant enzymatic activity potential in all six fermented maize‐based samples, primarily linked to enzymes involved in nucleic acid metabolism and amino acid biosynthesis, as highlighted in Figure 10. *Lactobacillus* was the predominant genus in the inferred enzymatic functions across all six samples, with varying contributions from other genera observed. *Lactobacillus* demonstrated notable abundance for EC3.6.4.12 (DNA helicase; 528.63) and EC2.7.7.7 (DNA‐directed DNA polymerase; 515.05) in S19 (fermented maize dough, artisanal), whereas *Weissella* was associated with EC1.1.1.1 (alcohol dehydrogenase; 53.10) and EC4.2.1.11 (enolase; 64.76). *Lactobacillus* was the main contributor in S37 (dehulled maize dough, commercial) across several significant enzyme commission numbers, including EC3.6.4.12 (679.21) and EC2.7.7.7 (582.11). *Streptococcus* played a role in EC2.7.7.7 (131.88) and EC5.4.2.2 (phosphoglucomutase; 90.63). S38 (12‐h fermented dehulled dough, commercial) demonstrated that *Lactobacillus* was the main predictor for EC3.6.4.12 (673.67) and EC2.7.7.7 (593.01), whereas *Streptococcus* contributed to EC2.7.7.7 (106.14) and *Acinetobacter* was linked to EC2.7.13.3 (histidine kinase; 23.62).

S4 (ogi, artisanal) exhibited a predominance of *Lactobacillus* in EC3.6.4.12 (685.61) and EC2.7.7.7 (589.23). *Acetobacter* participated in EC2.1.1.72 (site‐specific methyltransferase; 72.72) and EC2.7.1.1 (hexokinase; 43.32). S5 (12h‐fermented mawe, commercial) demonstrated increased levels of *Lactobacillus* in EC3.6.4.12 (854.50), EC2.7.7.7 (843.95), and EC2.7.1.1 (hexokinase; 794.35), while *Weissella* was associated with EC1.1.1.27 (l‐lactate dehydrogenase; 10.70) and EC2.7.1.1 (74.51). *Lactobacillus* was the predominant genus in S6 (48‐h fermented mawe, commercial) for the enzymes EC3.6.4.12 (851.45), EC2.7.7.7 (829.49), and EC2.7.1.1 (781.13). *Weissella* contributed to EC1.1.1.27 with a value of 10.54, whereas *Streptococcus* contributed to EC2.7.7.7 with a relative abundance of 69.14.

**Figure 11:**
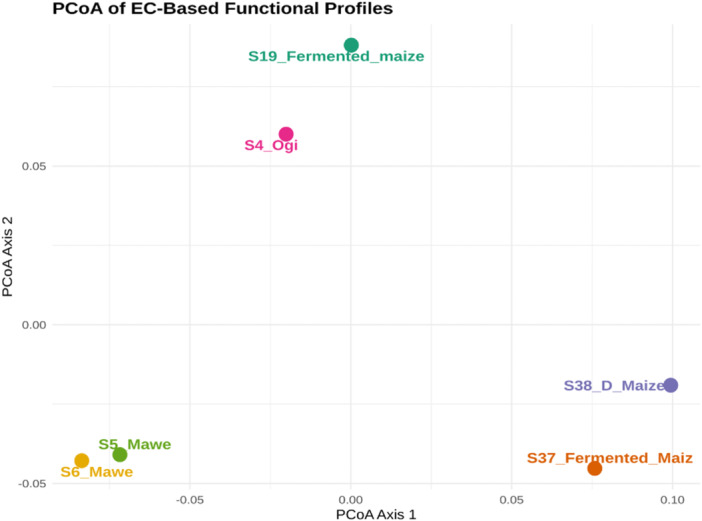
PCoA of Enzyme Commission (EC) contributions across samples.

Distinct clustering patterns were observed, indicating significant functional divergence across the samples (Figure 11). The S5_Mawe and S6_Mawe samples clustered closely. S19_Fermented_maize and S37_Fermented_Maiz samples were separated, with S19_Fermented_maize clustering away from S37_Fermented_Maiz, suggesting functional variation. Conversely, S38_D_Maize was distinctly isolated from all fermented samples, highlighting the pronounced effect of fermentation on the enzymatic functional landscape. S4_Ogi sample demonstrated a unique functional profile, distinct from the others.

#### 3.3.3 Functional Predictions at the KEGG Pathway Level

**Figure 12:**
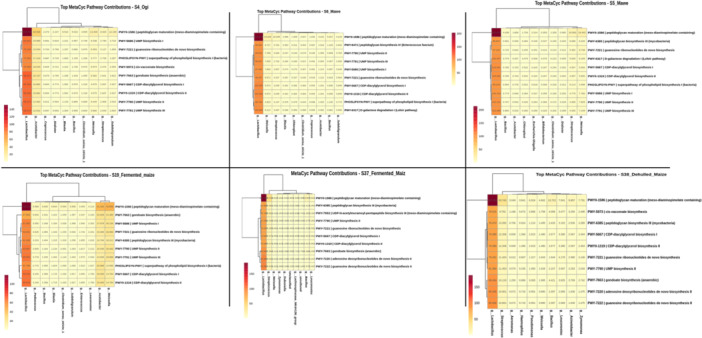
Combined heatmaps showing contributions of the top 10 bacterial genera to predicted metabolic pathways across six maize samples.

**Figure 13:**
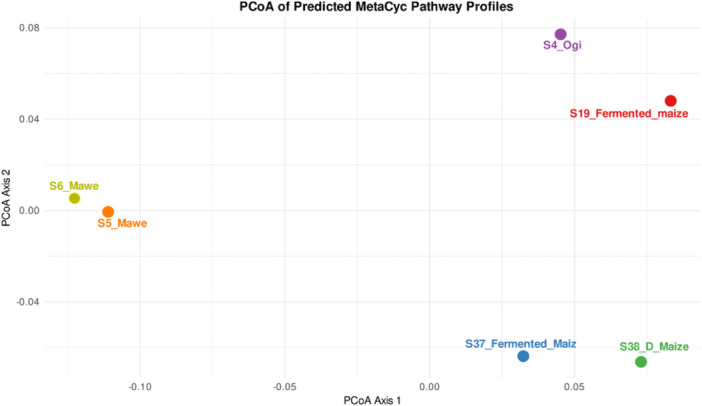
PCoA of predicted metabolic pathway contributions across samples.

The functional profiles at the pathway level, derived from PICRUSt2 analysis as illustrated in Figure 12, indicated a predominance of metabolic processes related to cell wall biosynthesis and nucleotide metabolism in the six fermented maize‐based food samples. Again, *Lactobacillus* was the main contributor to essential metabolic functions at the pathway level across all six samples, while other genera participated in specific biosynthetic pathways. In S19 (fermented maize dough, artisanal), *Lactobacillus* demonstrated the highest predicted abundance for PWY0‐1586 (peptidoglycan maturation; 119.12) and PWY‐6385 (peptidoglycan biosynthesis; 61.21). In contrast, *Weissella* was involved in PWY‐7663 (gondoate biosynthesis; 21.36) and PWY‐6385 (17.58). *Lactobacillus*‐dominated pathways in S37 (dehulled maize dough, commercial) included PWY0‐1586 (187.67) and PWY‐6385 (93.47). Conversely, *Streptococcus* contributed to PWY‐5667 (CDP‐diacylglycerol biosynthesis; 15.07) and PWY‐6385 (10.34). S38, a 12‐h fermented dehulled dough from commercial sources, demonstrated *Lactobacillus* as the dominant genus in PWY0‐1586 (187.38) and PWY‐7220 (adenosine deoxyribonucleotide biosynthesis; 90.44). In contrast, *Streptococcus* was associated with PWY‐5667 (12.21) and PWY‐6385 (8.43). S4 (ogi, artisanal) showed contributions from *Lactobacillus* to PWY0‐1586 (150.76), PWY‐6385 (83.45), and PWY‐5667 (85.95). In contrast, *Acetobacter* was involved in PHOSLIPSYN‐PWY (phospholipid biosynthesis; 17.42) and PWY‐6385 (11.03). S5 (12h‐fermented mawe, commercial) demonstrated *Lactobacillus* as the dominant genus in PWY0‐1586 (213.62), PWY‐6385 (121.09), and PWY‐5667 (103.98), while *Weissella* was present in PWY‐5667 (5.65) and PHOSLIPSYN‐PWY (3.47). In S6 (48h‐fermented mawe, commercial), *Lactobacillus* demonstrated higher values for PWY0‐1586 (203.05), PWY‐6385 (116.85), and PWY‐5667 (103.51), while *Weissella* contributed to PWY‐5667 (5.57) and PWY‐6385 (4.89). Unique predicted metabolic pathway contributions were also observed across all samples as shown in Figure 13.

## 4 Discussion

This study presents a comparative analysis of the microbial composition and predicted functional capacities of six traditional West African fermented maize‐based food samples. Using 16S rRNA gene sequencing and PICRUSt2‐based functional metagenomics, the findings indicate variation in microbial communities and their metabolic potentials, influenced by raw material processing, fermentation duration, and production practices (Shingling and Tamang 2021; Tamang et al. 2020; Ortiz et al. 2019).

### 4.1 Microbial Composition Across Phylum, Family, Genus, and Species Levels

At the phylum level, *Firmicutes* emerged as the predominant phylum across all samples, aligning with its recognized function in carbohydrate fermentation and organic acid production in lactic acid‐rich environments (Atter et al. 2021; de Jong et al. 2022; Zabat et al. 2018). As illustrated in Figure 2, their significant prevalence in S5_Mawe (*n* = 72,640; 94.9%) and S37_Fermented_Maize (*n* = 72,428; 91.4%) underscores the acidophilic characteristics of lactic acid bacteria (LAB) and corroborates earlier findings regarding the predominance of *Firmicutes* in cereal‐based fermentations (Srinivas et al. 2022; Yulandi et al. 2020). In contrast, *Proteobacteria* exhibited a greater relative abundance in S19_Fermented_Maize (*n* = 27,175; 36.5%), likely resulting from spontaneous fermentation with limited thermal treatment that favors opportunistic aerobic and facultative taxa (Navarro‐Díaz et al. 2022). The prevalence of *Actinobacteriota* across all samples suggests a stable background community that may play a role in the production of secondary metabolites (Varliero et al. 2023). *Lactobacillaceae* and the genus *Lactobacillus* were the predominant families and genera, particularly in S5_Mawe and S6_Mawe, as shown in Figure 4, highlighting their essential role in cereal fermentation (Palmnäs‐Bédard et al. 2023). These taxa are recognized for their acid production, proteolytic activity, and ability to competitively exclude spoilage organisms, as evidenced in various fermented food systems (Diwan et al. 2018). The elevated presence of *Acetobacteraceae* and *Acetobacter* in S19_Fermented_Maize suggests an oxidative microenvironment or extended fermentation conditions conducive to acetic acid bacteria (Oshiro et al. 2024). The identification of genera such as *Weissella*, *Zymomonas*, and *Acinetobacter* in specific samples indicates niche specialization; for instance, *Zymomonas* is known for its involvement in ethanol production during plant fermentations (Diaz et al. 2019). *Lactobacillus* fermentum was the dominant species in the fermented dough samples S37_Fermented_Maize (*n *= 10,585; 42.0%) and S38_Dehulled_Maize (*n* = 10,916; 42.3%) (Osuntokun et al. 2024). This species is associated with maize and cassava fermentations and is noted for its acid tolerance, rapid competitive growth, and probiotic benefits (Wang et al. 2019). The significant presence of *Lactobacillus* delbrueckii in S4_Ogi (*n* = 10,407; 40.3%) suggests an adaptation to heat‐treated substrates, as this species flourishes in thermophilic conditions commonly associated with cooking (Kim and Kim 2023). The microbial diversity identified in S5_Mawe and S6_Mawe, characterized by the presence of Leuconostoc, *Lactobacillus pontis*, and *Clostridium perfringens*, indicates a complex microbial succession pattern during fermentation (Hou et al. 2024).

### 4.2 Functional Inference With PICRUSt2

#### 4.2.1 Taxon Contributions to KOs

Our study shows that *Lactobacillus* is the main contributor to the functional potential of most predicted KOs in all samples. As shown in Figure 6, samples S19 maize dough and S4_Ogi show a strong dominance of *Lactobacillus* in pathways such as K02029 (polar amino acid transport permease) and K02529 (LacI transcriptional regulator) (Adesulu‐Dahunsi et al. 2022; Kharnaior et al. 2020). *Weissella* is linked to transport functions, including K01990, the ABC‐2 type transport system ATP‐binding protein, and K02035, the peptide/nickel transport system substrate‐binding protein, in specific commercial samples (Liu et al. 2025). *Streptococcus* plays a role in ABC transport (K06147) in samples S37 and S38, suggesting a sample‐specific functional contribution that is consistent with earlier research on cereal fermentation, which highlights the impact of minor genera on metabolic outputs (Phiri et al. 2019). The minor contributions from *Acetobacter* in sample S4 indicate a diversified microbial consortium that may influence accessory functions, including redox balance and flavor development (Oyeyipo et al. 2020). The associations between taxa and functions correspond with earlier studies on African indigenous foods, indicating that a primary group of lactic acid bacteria facilitates fermentation and improves functional and safety characteristics (Adesulu‐Dahunsi et al. 2022; Obafemi et al. 2022). The involvement of *Lactobacillus* in transposase activity (K07496) in samples S5 and S6 suggests genomic plasticity and adaptation to fermentation environments (Duffy et al. 2015).

Previous studies on African fermented cereal‐based products indicate a predominance of lactic acid bacteria. Oyeyipo et al. (2020) found that co‐fermentation of maize with African breadfruit seed led to microbial profiles predominantly consisting of beneficial LAB, corroborating our findings in artisanal Ogi products (Adesulu‐Dahunsi et al. 2022). Research on commercially processed products indicates heightened contributions from genera such as *Streptococcus* in maize dough products, paralleling our S37 and S38 profiles (Liu et al. 2025). Additional studies indicate that functional properties, such as transport systems and regulatory proteins, are associated with enhanced nutritional quality and overall food safety, a trend reflected in our findings (Adesanmi et al. 2020; Mohammed et al. 2021). In comparison to fermented cereal products beyond Africa, such as sourdough breads (Felenou and Elisee 2019) and fermented bean products (Liu et al. 2020), the persistent presence of *Lactobacillus* and other lactic acid bacteria as essential functional agents reinforces the notion of conserved microbial functionality across various substrates (Obafemi et al. 2022). Non‐LAB taxa, such as *Weissella*, exhibit strain‐specific metabolic capabilities that warrant further targeted investigations (Liu et al. 2025). The ecological principles of fermentation are illustrated through the contrasts and similarities present in the cultural and processing variables that influence maize‐based products (Hawaz et al. 2025).

*Lactobacillus* synthesizes lactic acid and antimicrobial substances, enhancing the safety of fermented maize products through pH reduction and the inhibition of spoilage organisms (Galati et al. 2014; Özogul and Hamed 2017). The associations with K02029 and K02529 suggest that amino acid transport could enhance nutrient assimilation and improve the nutritional value of the product (Ijarotimi 2022). The presence of transporters such as K01990 signifies metabolic activity that facilitates energy expenditure for cellular maintenance in fermentation environments, consistent with findings from food studies (Adeoti and Osundahunsi 2017). Genera such as *Weissella* and *Streptococcus* contribute to flavor profiles and textural properties, which may impact consumer preference and shelf life (Sawant et al. 2025). The findings corroborate previous studies linking microbial activity to enhanced bioavailability of vitamins and peptides, thereby improving the functional and health‐promoting properties of fermented foods (Ashaolu 2019; Capozzi et al. 2020). The identification of sequences associated with transposase activity (K07496) indicates possible genomic rearrangements that could facilitate the adaptation of microbial communities and enhance the fermentation process, thereby ensuring safety and nutritional outcomes (Duffy et al. 2015).

#### 4.2.2 Taxonomic Contributions to Enzyme Commission Predictions

Our results, as presented in Figure 8, show that *Lactobacillus* is a key contributor to enzymatic functions, indicated by its strong association with EC3.6.4.12 (DNA helicase) and EC2.7.7.7 (DNA‐directed DNA polymerase) in all samples, particularly in artisanal products (S19 maize dough, S4 Ogi) and commercial products(S37 maize dough, S38 maize dough, S5 Mawe, S6 Mawe) (Adesulu‐Dahunsi et al. 2022; Zhang et al. 2023). This role reinforces the capacity of *Lactobacillus* to drive replication and metabolic renewal during fermentation, ensuring high cellular processes and resilience (Adesulu‐Dahunsi et al. 2022). Other genera, like *Weissella* and *Streptococcus*, were associated with specific enzymes. For example, *Weissella* contributed to EC1.1.1.1 (alcohol dehydrogenase) and EC1.1.1.27 (L‐lactate dehydrogenase), indicating its role in redox balancing and flavor development (Ashaolu 2019; Kothe et al. 2024; Singh et al. 2024). *Streptococcus* contributed to EC5.4.2.2 (phosphoglucomutase) and was also associated with EC2.7.7.7, highlighting its involvement in carbohydrate metabolism and energy regulation (Adesulu‐Dahunsi et al. 2022). Minor contributions from *Acetobacter*, *Acinetobacter*, and other taxa indicate a diverse ecosystem that performs primary metabolic functions and accessory reactions essential for product safety and quality (Adesulu‐Dahunsi et al. 2022). Taxon–enzyme function contributions correspond with observations in other African fermented systems, wherein microbial interactions facilitate safety and the production of bioactive metabolites (Obafemi et al. 2022).

A comparative assessment shows that our findings align with recent studies characterizing the metabolic profiles in fermented foods. Han et al. (2024) demonstrated that the inoculation of lactic acid bacteria alters the enzymatic profile during fermentation, marked by a predominance of enzymes related to replication and metabolism, as observed by the significant presence of EC3.6.4.12 and EC2.7.7.7 in our samples. Gangakhedkar et al. (2025) highlighted the importance of enzyme systems in traditional fruit and vegetable fermentations, which improve functional and sensory characteristics; these results align with the enzymatic profiles identified in maize dough and Ogi samples in this study. Taale et al. (2024) found that microbial consortia from indigenous West African fermentations show similar taxonomic patterns, primarily driven by *Lactobacillus*, while also noting contributions from *Weissella* and *Streptococcus*. Research by Ugbogu and Okereke (2023) on Bambara flour fermentations, along with studies by Phiri et al. (2019) on cereal‐based beverages, has highlighted the involvement of non‐*Lactobacillus* taxa in enzyme‐mediated carbohydrate conversion, thereby supporting our findings.

The enzymatic activities predicted through the PICRUSt2 analysis bear significance for food safety and nutritional quality. The anticipated concentrations of EC3.6.4.12 (DNA helicase) and EC2.7.7.7 (DNA‐directed DNA polymerase) suggest ongoing cell division and repair processes that enhance the stability of the fermenting community and facilitate the elimination of damaged cells (Zhang et al. 2023; Borrego‐Ruiz et al. 2024). Enhanced replication machinery is essential for suppressing spoilage organisms through rapid acidification and organic acid production, thereby ensuring product safety (Borrego‐Ruiz et al. 2024). Enzymes such as EC1.1.1.1 and EC1.1.1.27 from *Weissella* play a role in producing flavor‐active compounds and maintaining redox potential, thereby enhancing the sensory qualities and consumer acceptance of traditional foods (Anumudu et al. 2024). *Streptococcus* and other minor genera contributed to carbohydrate metabolism via phosphoglucomutase (EC5.4.2.2), facilitating energy transfer and enhancing the bioavailability of nutrients such as vitamins and amino acids (Tsafrakidou et al. 2020). The enzyme profiles indicate that the microbial community in maize fermentation is organized to enhance safety and nutrition, thereby augmenting the functional and health‐promoting potential of these products (Capozzi et al. 2017; González‐González et al. 2022).

#### 4.2.3 Insights into Functional Contributions and Pathway Profiles by Taxon

As represented in Figure 12, all six samples showed a significant contribution from *Lactobacillus* in several pathways within the MetaCyc database, especially in cell wall processes such as peptidoglycan maturation (PWY0‐1586) and peptidoglycan biosynthesis (PWY‐6385) (Adesulu‐Dahunsi et al. 2022; Phiri et al. 2019). Our taxon–function contributions align with findings in maize dough fermentations for Pozol and Masa, where lactic acid bacteria control biosynthetic pathways essential for cell integrity and community regeneration (Duffy et al. 2015; Li et al. 2025; Li et al. 2025; Phiri et al. 2019). Comparisons with fermented cereal products from various regions highlight the importance of these functional contributions. The Mexican Pozol (Duffy et al. 2015; Phiri et al. 2019) and South African Munkoyo beverages (Mohammed et al. 2021) show that peptidoglycan maturation and biosynthesis are common features of lactic acid bacteria‐dominated fermentations. *Lactobacillus* predominates in our maize‐based fermentations, consistent with findings from Doklu and other maize products(Ijarotimi 2022), where cell wall integrity relates to fermentation stability and acidification (Han et al. 2024). The involvement of *Weissella* and *Streptococcus* in lipid and nucleotide pathways like PWY‐5667 and PWY‐7220 has been noted in other cereal‐based fermentations, where these genera contribute to flavor development and membrane remodeling (Oyeyipo et al. 2020). Furthermore, metabolic profiling of maize products in our study contributes to the significance of CDP‐diacylglycerol biosynthesis and associated lipid metabolic pathways in microbial adaptation to fermentation stresses (Adesanmi et al. 2020; Felenou and Elisee 2019). The convergence of metabolic predictions in these studies supports our findings and demonstrates the role of lactic acid bacteria in influencing fermentation outcomes in various cereal substrates (Galati et al. 2014).

The predicted functional profiles have consequences for food safety and nutritional quality. Elevated levels of pathways such as PWY0‐1586 and PWY‐6385 suggest active processes of cell wall synthesis and maturation in *Lactobacillus*, which are critical for microbial growth and the competitive exclusion of pathogens (Han et al. 2024). The function of CDP‐diacylglycerol biosynthesis (PWY‐5667) in membrane lipid assembly, facilitated by *Streptococcus* and supported by *Lactobacillus*, may contribute to bacterial resilience in low‐pH environments, which is a critical safety feature in spontaneous fermentations (Adeoti and Osundahunsi 2017). Adenosine deoxyribonucleotide biosynthesis (PWY‐7220) facilitates nucleic acid regeneration and genomic plasticity, which are crucial for adaptive responses to fermentation stress (Sawant et al. 2025). The participation of minor taxa like *Acetobacter* in phospholipid biosynthesis (PHOSLIPSYN‐PWY) suggests roles in flavor modulation and enhancement of bioactive compounds important for nutritional enrichment (Mohammed et al. 2021).

### 4.3 Future Directions in Metabolic Profiling of Fermented Maize Products

To advance beyond the predictive functional insights generated in this study, future research must prioritize the experimental validation of key metabolic pathways through an integrated multi‐omics framework. While this study highlighted the potential of dominant genera such as *Lactobacillus*, *Weissella*, and *Streptococcus*, metatranscriptomic analysis is explicitly required to distinguish between the presence of functional genes and their active expression during critical fermentation stages. This approach would clarify whether the predicted pathways for peptidoglycan biosynthesis, lipid metabolism, and ABC transport are actively driving community dynamics or merely present in the genomic background (Duffy et al. 2015; Plichta et al. 2016). Furthermore, targeted metabolomics should be employed to correlate the predicted enzymatic activities with tangible biochemical end‐products, particularly focusing on the quantification of flavor volatiles, organic acids, and anti‐nutrient reduction markers predicted by these models (Haripriyaa et al. 2025). Such data is essential for designing rational synthetic consortia. Rather than generic starter cultures, future strategies should isolate and recombine the specific Lactobacillus and Acetobacter strains identified here to create defined functional guilds that ensure consistent product outcomes and enhance safety (Dhiman et al. 2025; Irakoze 2024; Nissen et al. 2019). Finally, linking these molecular mechanisms to phenotypic outcomes such as specific vitamin production profiles or toxin degradation rates will allow for the precise regulation of fermentation parameters (Kårlund et al. 2020; Sawant et al. 2025; Zannou et al. 2023). This transition from predictive profiling to mechanistic validation is crucial for the production of reliable and safe maize‐based foods (García Méndez et al. 2021; Pei et al. 2022; Tamang et al. 2020; Capozzi et al. 2020; Ashaolu 2019).

## 5 Conclusion

The predicted functional landscape of traditional maize‐based fermented foods shows a microbiome primarily shaped by LAB with enhanced genomic potential for carbohydrate processing, amino acid assimilation, DNA replication, and cell wall biosynthesis. The differences in functional gene content across raw and cooked maize dough products indicate ecological specialization influenced by fermentation regimes and substrate processing. However, we acknowledge that these insights are derived from predictive modeling (PICRUSt2) using 16S rRNA taxonomic markers on a limited sample set. Consequently, the metabolic pathways described here should be interpreted as high‐confidence hypotheses regarding the functional potential of these communities, rather than experimentally validated enzymatic mechanisms. Despite these methodological limitations, these findings provide a good baseline for future multi‐omics validation and present an opportunity for bio‐enriching these products through microbial management and controlled fermentations.

## Author Contributions

**Humphrey P. K. Addy:** conceptualization, methodology, data curation, formal analysis, visualization, writing‐ original draft, writing – review and editing. **David Amedorme:** methodology, data curation, formal analysis, writing – review and editing. **Priscilla Osei‐Poku:** conceptualization, methodology, project administration, writing – review and editing. **Alexander Kwarteng:** conceptualization, methodology, project administration, supervision, writing‐ review and editing.

## Funding

The authors received no specific funding for this work.

## Ethics Statement

The authors have nothing to report.

## Conflicts of Interest

The authors declare no conflicts of interest.

## Supporting information

Supplementary material.

## Data Availability

The data that support the findings of this study are available in Sequence Read Archive (SRA) at https://www.ncbi.nlm.nih.gov/sra/?term=PRJNA532858, reference number PRJNA532858. These data were derived from the following resources available in the public domain: https://www.ncbi.nlm.nih.gov/bioproject/PRJNA532858. Code Availability: All the codes or scripts used for this functional metagenomics analysis using 16S data is available and can be accessed on GitHub via https://github.com/humphreyaddy/16S_microbiome_analysis_MPhil-BACG-project.
